# Expanding the role of midwives in Korea

**DOI:** 10.4069/kjwhn.2021.09.09.1

**Published:** 2021-09-30

**Authors:** Kyung Won Kim

**Affiliations:** Department of Nursing, Daegu Haany University, Daegu, Korea

According to the State of the World’s Midwifery 2021 report, there are about 1.9 million midwives worldwide, but a shortage of 900,000 midwives remains. The workforce of midwives is insufficient in European countries, the United States, Australia, and Japan, where midwives help with childbirth, as well as in Africa, where the maternal mortality rate is high [1].

In Korea, where 99.5% of newborns are born in hospitals, midwives are also facing a crisis due to the deteriorating circumstances in the field of obstetrics and gynecology resulting from the ultra-low birth rate [2]. As apprenticeships for midwives have disappeared, it has become difficult to train midwives. Midwives are also losing their jobs and closing birth centers in response to the decreasing number of midwife-assisted births, after an earlier period of growth due to the popularity of natural childbirth. Accordingly, this article was written to shed light on the role of midwives during the transition process of the Korean midwifery system and to explore ways of broadening the role of Korean midwives in the future by analyzing examples of the expansion of midwives’ role in other countries.

## Midwifery policy in Korea

The profession of midwifery in Korea began with the introduction of Western medicine in the early 20th century. In the past, there were “three-grandmothers (*samshin*) ” and “umbilical cord-cutting grandmothers,” who simply helped give birth and cut the umbilical cord. However, as their actions were considered superstitious and unsanitary, lay midwives (*sanpa*) appeared. Unlike the traditional “grandmothers,” midwives (*sanpa*) acted as mothers’ assistants, helping with prenatal and postpartum management and childbirth, as well as checking the infant's health [3]. The term for midwives (*sanpa*) was changed to *josanwon* in 1951 and *josansa* in 1987 [4].

During the Japanese colonial period, a midwife’s license could be obtained after completing a course at a midwife training center. After Korea's liberation, graduates from nursing school were also eligible to get a midwifery license. Those who did not attend a midwife training center or nursing school could take a midwife qualification examination, but this examination was difficult to pass [3]. For nursing school graduates, the nursing-midwife policy was implemented, allowing nurses to obtain a midwife's license if they assisted 20 childbirths [4]. This policy enabled nurses who received nursing education to become midwives upon completion of midwifery training. This reflected the American system, wherein midwives contributed to lowering maternal and infant mortality by engaging in childbirth-related work. These goals were also pursued by the U.S. Military Government. [5].

This system continues to this day. Today, midwives in Korea must pass the midwives’ national examination after completing a 1-year midwifery probationary course after holding a nurse's license [6]. Midwives are medical personnel and nurse midwives, and their role is greater than that of traditional midwives and nurses separately.

## The role of midwives

The Medical Service Act of Korea states that midwives are tasked with providing health and health guidance for childbirth, pregnant women, and newborns [6]. Midwives are mainly responsible for childbirth-related tasks, but in the 1950s, they were also responsible for managing infectious diseases and basic health care, as well as checking population-level dynamics such as population movements, deaths, and births due to the Korean War. Midwives in Korea have consistently performed midwifery duties, but the midwifery probationary course was operated as a special hospital course; therefore, the curriculum and midwives’ roles were not sufficiently evaluated. In 2003, core competencies were developed through a midwifery work analysis [7]. More recently, a midwifery work guideline was developed for midwives and has been made available to the public to promote a wider understanding of the actual conditions of midwives’ jobs. According to the work guidelines for on-site midwives, midwives perform 56 tasks in 7 areas: pregnancy management, childbirth management, postpartum management, newborn care, primary health care, law/ethics, and general management [8].

The role of midwives in pregnancy management has focused on pregnancy discomfort management, self-care education/counseling, physical assessment, fetal assessment, and history taking. In delivery management, their role involves caring for the first, second, third, and fourth stages of childbirth and pain management. Postpartum care includes postpartum assessment, postpartum home visits, maternal education, maternal self-care, breastfeeding, and newborn care (including both newborn health assessment and normal newborn care) [7,8]. Primary health care encompasses women’s health management, counseling, and education. Furthermore, midwives serve as women's health professional nurses capable of assessing women’s health.

Midwives have faced changes in their role and an occupational crisis as hospital treatment and hospital births surged in Korea [9], starting with the implementation of the national health insurance system. This is related to the expansion of analgesia-assisted labor through hospital births and the implementation of high-risk pregnancy management. However, as women complained about impersonal treatment and excessive medical interventions in the hospital birth process, women themselves reclaimed bodily agency and searched for natural childbirth methods that enable them to experience childbirth as active participants, rather than as passive subjects of medical treatment [9]. For this reason, births in birthing centers and at home increased, and midwives re-emerged as facilitators of childbirth. As midwives applied alternative delivery methods preferred by women, their roles in the existing 7 areas expanded and changed. Midwives help women to accept labor as a physiological process and to have natural childbirths with minimal medical intervention. There is a need for education for women who want to give birth in birthing centers, as well as for their family members, and strengthening midwifery plays a meaningful role in supporting these initiatives [10].

Recently, however, Korean midwives are once again facing a crisis involving role change. Due to women's preference for natural and self-directed childbirth, the number of birthing centers in Korea increased from 13 in 2012 to 20 in 2014, and the number of childbirths at these centers also reached 1,679 in 2013 [11]. However, as the total fertility rate fell to 1.05 in 2017 and 0.92 in 2019, the number of birthing centerss dropped to 15 in 2019, and the number of natural births at these centers decreased by nearly 1,000 to 683 [12]. As midwives’ role has been mainly focused on childbirth and health care for mothers and infants, the falling birth rate has made it difficult for midwifery to be sustainable as a medical profession in Korea. This underscores the need to expand the role of midwives.

## Expansion of midwifery roles in in other countries

As Korea’s low total fertility rate has threatened midwives’ position, it is meaningful to explore steps taken by several other countries that have already experienced a drop in the fertility rate, to expand the role of midwives by predicting relevant sociodemographic shifts.

In Sweden, midwives are in charge of childbirth in the hospital delivery room, and midwifery visit centers provide prenatal care including ultrasonography, health assessments, and counseling on exercise and nutrition. Maternity centers provide 1-day postpartum management, examinations at 12 to 14 weeks postpartum, contraceptive management, and postpartum management until 16 weeks (vaginal observations, checking for infections of the surgical wound after cesarean section, wound dressing managing, breastfeeding-related problems, assessing and managing postpartum depression, etc.). In addition, midwives in Sweden perform a wide range of roles, including education on parenting roles and family planning, health information education, sexual education, contraceptive education, counseling for mental disorders, and counseling on school life maladjustment for elementary school students and adolescents [13].

Midwives in the United Kingdom not only care for mothers and infants, but also manage health problems such as domestic violence, sexual violence, mental illness, and substance abuse. Midwives visit homes to monitor children’s health and safety. The level of parenting skills is identified and, if an intervention is required, linkage is provided to related institutions [14].

Midwives in Canada provide pregnancy and childcare services as well as childbirth support, promote information exchange among mothers and link mothers to services within the local community [15].

In Turkey, in addition to their role in reducing maternal and infant mortality and improving health, midwives are responsible for the health of women, infants, families and communities through a scientific process that meets modern standards in collaboration with perinatal health experts [16].

Midwives in the United States practice health promotion and disease prevention while focusing on women’s primary health care for each stage in the life cycle. The work of midwives reflects changing social needs and promotes the development of knowledge through research activities [7].

In these countries, the recipients of health care provided by midwives have expanded to include all members of the community, from mothers and infants to adolescents and adults. In addition to childbirth management, midwives maintain their professional role as medical professionals by expanding the scope of their work to include that of professional nurses, encompassing the physical and mental health of community members, sexual health education and counseling, and domestic violence and substance abuse management. Moreover, the state provides a medical system in which midwives can perform professional roles while achieving the standardization of midwifery work.

## Directions for midwives' role expansion in Korea

Korean midwives are also seeking ways to expand their role in response to changes in childbirth patterns. First, by providing childbirth assistance to families who need midwives, they fulfill the original role of midwives stipulated in the Medical Services Act. Midwives can be in charge of prenatal and postpartum management of women in vulnerable regions, such as rural areas and fishing villages, where there is no obstetrician or gynecologist, even if a delivery room exists. The State of the World’s Midwifery 2021 report [1] noted that midwives are equipped to play a pivotal role in achieving the Sustainable Development Goals set by the United Nations. This is also consistent with the expanded role that Korean midwives are currently seeking to undertake. Second, efforts are being made to expand the level of health care, education and counseling, and health assessments, which midwives mainly provided for women, to a broader range of individuals, families, and community members. In other words, midwives can become more active in promoting health and participating in primary health care that provides integrated services for disease prevention, treatment, and rehabilitation. In addition, midwives can carry out health screening, vaccination, and preventive education and management performed by nurses in the current coronavirus disease 2019 pandemic. This is possible because Korean midwives are essentially nurses with midwifery training. Korean midwives can conduct maternal and child health programs at public health centers at the municipal and county level in the local community and are well poised to provide family planning services. Moreover, midwives have the capacity to perform the role of primary health managers by providing regular health checkups, vaccinations, and home care for pregnant women and infants.

Given that the role of midwives is expanded, people will more easily recognize midwives as medical professionals responsible for women's health care and the health of community residents. Such recognition will in turn, allow midwives to build on their rich history of contributing to maternal child health, to establish a clearer position and stronger role for promoting people's health in Korea.

## References

[1] http://www.unfpa.org/sowmy.

[2] http://www.wedd.tv/news/articleView.html?idxno=3928.

[3] Lee IH (2016). Training of midwife as female professional in 1950s in Korea. Soc Hist.

[4] Park HS, Kim YS, Chung SM (2017). Exploring the meaning of social practice in the midwife life history. J Educ Cult.

[5] Lee SJ (2008). Japanese American Midwives: Culture, Community, and Health Politics, 1880-1950 (University of Illinois Press, 2005). Yonsei J Med Hist.

[6] https://www.law.go.kr/LSW//lsSc.do?section=&menuId=1&subMenuId=15&tabMenuId=81&eventGubun=060101&query=%EC%9D%98%EB%A3%8C%EB%B2%95#undefined.

[7] Lee KH, Kim KW (2003). Core competency of basic practice of nurse-midwifery. Korean J Women Health Nurs.

[8] Song JY, Park YJ (2020). Opening status of the Korea midwifery birthing centers and development of midwifery practice guideline. J Korean Acad Nurs.

[9] Park JM, Park HR (2019). Development and effect of nurse-centered doula support program for mothers with natural childbirth: pilot test. J Korean Soc Matern Child Health.

[10] Park JS, Kwon YE, Kim BM (2017). The job experience of nurse midwives. Korean J Health Commun.

[11] https://kosis.kr/statHtml/statHtml.do?orgId=354&tblId=DT_LEE_54&vw_cd=MT_ZTITLE&list_id=354_1_F&scrId=&seqNo=&lang_mode=ko&obj_var_id=&itm_id=&conn_path=E1.

[12] http://www.index.go.kr/potal/main/EachDtlPageDetail.do?idx_cd=1428.

[13] Shin JH (2016). A comparative study on midwifery: Korea and Sweden. Korean J Women Health.

[14] Lee NH (2014). Ways to improve prenatal care in a low-fertility Korea. Health Welf Policy Forum.

[15] Lee YS, Park JY, Kim HJ (2015). A field study of 「Toronto Birth Centre」 in the perspective of a public service strategy coping with low birth-rate. J Korean Hous Assoc.

[16] Vermeulen J, Luyben A, O’Connell R, Gillen P, Escuriet R, Fleming V (2019). Failure or progress?: The current state of the professionalisation of midwifery in Europe. Eur J Midwifery.

